# Promotion and sustainable development of beef cattle farming industry in agro-pasture ecotone areas, Inner Mongolia of China: A comparison between two fattening systems

**DOI:** 10.1016/j.heliyon.2022.e12721

**Published:** 2023-01-02

**Authors:** Yujie Liu, Muhammad Umer Arshad, Baoyindureng, Aruhan, Régis Lanneau, Yang Jianguo

**Affiliations:** a Inner Mongolia Agricultural University, China; b Inner Mongolia Honder College Arts and Sciences, Hohhot, China; c Inner Mongolia Normal University, Inner Mongolia, Hohhot, China; d University of Paris Nanterre, CRDP, France

**Keywords:** Agro-pasture ecotone, Two fattening approaches, Cost-benefit analysis, Beef cattle farming

## Abstract

The agro-pasture ecotone is distributed all around the world. In these areas, the productive land forces are decreasing, and due to the irrational economic activities and the vulnerable ecological environment in these regions occurs land degradation. This study focuses on the effect of two different fattening approaches of beef cattle and output from the economic point of view by using a cost-benefit analysis technique in the eastern agro-pasture ecotone of Inner Mongolia, China. This study considers the environmental, social, and economic costs as input factors and concludes that both fattening systems have different characteristics. The result shows that the intensive farming system has more fluctuation and instability in terms of the number of animals due to the feed shortage in the local area. In comparison, the continuous fattening system is much more efficient and sustainable in terms of cost management and benefit analysis due to the local condition of the area. The empirical results indicate that the beef cattle industry has a high marginal return. Our research highlights the need to prioritize local resources and incorporate feed-intensity analysis in livestock.

## 1 Introduction

The productive land forces and animal husbandry are reducing in agro-pasture ecotone (APE) areas due to the excessive population density, vulnerable ecological environment, and complex household economy structure; consequently, land degradation in these regions can be significant [1]. In the agro-pasture ecotone, sustainable agriculture development has been long concerned with the development of the regional economy and environmental protection. In most countries, the development of these areas is crucial for ecological and economic development. Due to the restriction of environmental conditions, agriculture projects' efficient and sustainable development has become important for the local agricultural industries.

China has one of the world's distinct agro-pasture ecotone (APE) areas covering 129,600 km^2^ of land in the North, bounded by 35–50°N and 100–125°E [2,3]. With a population of 67 million, nearly 85% of natural grassland of the APE suffers from degradation. Therefore, the sustainable development of the local agricultural industry is substantial. In China, the agro-pasture ecotone is distributed in Liaoning, Jilin, Hebei, Shanxi provinces, Ningxia, and Inner Mongolia Region [4, 5].

### 1.1 Beef production and Inner Mongolia, Tongliao case

Tongliao is located in the Northeast of the Inner Mongolia Autonomous region of China with a population of 2.86 million. In Tongliao, almost every household is involved in the beef cattle farming business. Thousands of small-size farms coupling crop planting and cattle feed cultivation have formed a beef cattle farming industry on a considerably large scale. The small household brings many advantages to the local farming industry, and the loss is minimized to a smaller size. Small farms also adopt more flexible farming and management methods suitable for the local natural and social conditions. The risk of large-scale farming has been broken down into small pieces during the epidemic of the animal plague.

According to different farming conditions, two main paths of beef cattle farming are adopted in Tongliao agro-pasture ecotone (Tongliao APE). The first path is the traditional herding system (continuous fattening system). In this system, the local farmers follow the conventional approach in the breeding and farming process. Shelf cattle fattening production cycle is six to ten months, while the production cycle for breeding cows is two years. By increasing the number of cattle breeding and selling, the capital saving of each household is increased. This system needs more herding lands for feed supply and cultivation skills.

The second path is the modern concentrated fattening style which follows the yard fattening approach. Farmers purchase the feeder cattle from the livestock market, then raise them for about 12 months (intensive fattening) in their fattening yard to increase the weight of beef cattle and sell them to the cattle agents or companies#. The second path requires less land, and the farming cycle is shorter. However, it requires more technology and capital input for feeding and veterinary health care during the fattening cycle. According to survey data, about 70% of farmers are adopting the first path, and 30% of local farmers are using the second path for cattle farming development in Tongliao area. The first system is adopted by villagers such as the Galadaqi have more herding lands and abundant labour supply. The villages such as Haolibao, which has less land, but more capital stores, adopt the second fattening system. Tongliao's beef cattle farming industry is the meeting point of "traditional" and "modernization" in the animal husbandry of agro-pasture ecotone (APE).

**Fig. 1:**
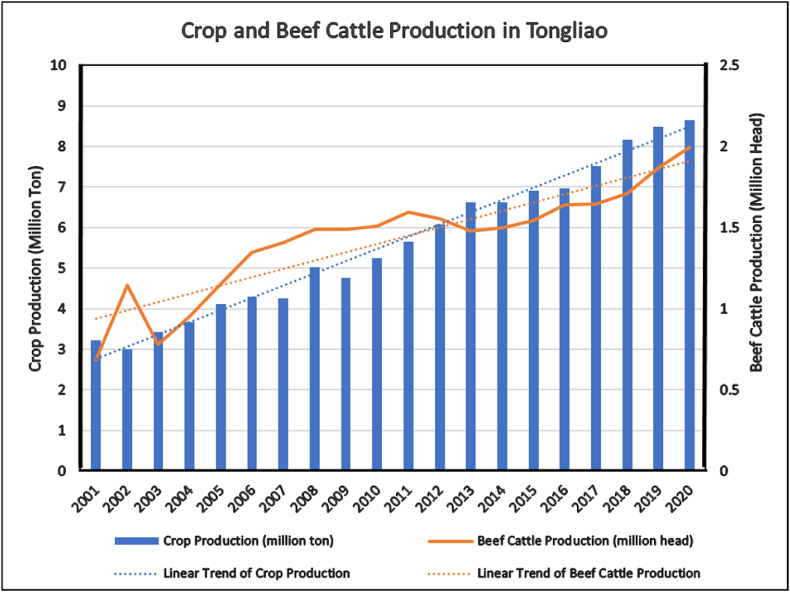
Crop and beef cattle Production in Tongliao (statistic Yearbook of Inner Mongolia 2002–2021).

Tongliao has developed the beef cattle farming industry as the leading agricultural sector contributing 20% to the local GDP. The total cultivated land is 1.4 million hectares. The sown area of grain crops is 1.23 million hectares. In 2019, the crop output was 8.486 million tons. The average yield of grain crops was 13,770 kg/ha, 3.4% higher than the previous year. The annual livestock stock in animal husbandry was 9.0725 million, a 4.1% increase from a year earlier. Animal husbandry includes 2.4169 million cattle, 5.33 million sheep, and 1.915 million pigs [7]. In the last decade, an extensive supply of feed resources accumulated in traditional experiences of beef cattle farming by utilising the ideal local conditions for the beef cattle industry and relatively abundant labour resources. From 2010 to 2020, the stock of beef cattle increased from about 1.9 million to 3.3 million in Tongliao, achieving a surge of 73.6% [8]. Fig. 1 represents the crop planting and beef cattle farming data in the last two decades in Tongliao.

The beef cattle industry is an essential agricultural sector in the region. The beef cattle industry has benefited the local economic and social development in the last decade, as the agriculture industry of Tongliao will contribute 25.8% to the GDP of 2021. In the long run, APE's requirements are getting much more difficult for sustainable and efficient agricultural projects. Due to the environmental welfare restriction, shortages in fattening could eventually hamper the industry's sustainable development.

A balance between the cost and benefit of the sustainable livestock sector has become crucial as the production and consumption of livestock products are more strictly examined on environmental welfare grounds [[9], [10], [11], [12]]. Previous research pointed out that intensification and the modernization of the animal husbandry industry effectively reduce the pressure on the environment [[13], [14], [15], [16], [17], [18]]. Intensive fattening is a better option to reduce emissions and waste dumping in the environment, preventing climate change, less use of land, greenhouse gas emission reduction, and grassland degradation [19,20]. The analysis of net present values (net profit), opportunity costs, and sensitivity proved that the intensive fattening system is more efficient [13,[21], [22], [23], [24]]. Meanwhile, the intensive fattening system also has high economic efficiency in production, supply, and distribution [21,25,26]. In China, the remote fattening mode as the industrial division and specialisation of calf breeding, feeder cattle raising, and intensive fattening with complementary resource advantages in different areas has promoted the economic efficiency of the whole industry [27,28].

The intensive fattening system has been proven to have certain drawbacks. For example, the carbon efficiency of the hybrid form has been tested out that more optimal than the intensive fattening system when in a continuous fattening system, agricultural projects were mixed with small-large livestock breeding and product-oriented management[29, 30]. An intensive fattening system requires higher technology adaptation and better feed supply. The economic efficiency is hampered by low technological accumulation and an insufficient supply of feed and water areas [31]. The feed utilisation rate for intensive fattening is crucial, but it has been calculated as less than the continuous fattening in Tongliao, Inner Mongolia [3]. Farm size plays a vital role in the outcomes of adopting two systems. The feasibility of adopting the intensive fattening system is much rare in groups of small farms [32,33].

The continuous fattening system is advantageous for economic and social sustainability in Inner Mongolia [32]. Traditional and semi-herding techniques have a high return on investment without adopting high technology and automation systems [34]. A massive feed supply in the local pasture and fewer transportation costs increase feed utilisation [32]. In addition, the pressure of the continuous fattening system on manure treatment is less than the intensive fattening system [6]. Local farmers are also involved with the concurrent business because the corn planting provides feed. The economic efficiency of continuous fattening could be higher than intensive fattening in normal years [35]. From the viewpoint of cost-benefit analysis, the continuous fattening system has its merit in cost, which considers the economic outcomes. Although the economic merit could achieve certain gains, the farming cycle of the continuous fattening system is almost twice as compared to the intensive fattening system. The higher economic risk and long waste dumping period may affect the performance of sustainability of the continuous fattening system.

Although massive research has been conducted comparing continuous and intensive fattening systems in description and qualitative analysis in animal husbandry management. In contrast, few studies applied economic calculation to zoom in on the advantages and disadvantages of two fattening systems in Inner Mongolia agro-pasture ecotone. The environment's cost for using these two systems was not fully estimated. Suggestions to improve the structures and process of two fattening systems for sustainable development are limited. More research must consider the practical problems of both fattening systems in relatively low beef productivity. The surging cost of cattle raising, lack of training and education on cattle farming, and difficulties in waste treatment are the main bottlenecks for beef cattle farming in Tongliao. Filling the research gap on the sustainable development of the agriculture industry and improving the social life in the APE area could be urgent for the scientific development of the local beef cattle industry. With the above background, this research aims to qualitatively compare the performance of two systems on sustainable development in the view of economic efficiency by including the environmental cost. Moreover, suggestions for improving the sustainable development of these two systems under the unique condition of Tongliao APE are studied in this paper. Section 2 will present the research method, data, and variables of this study; section 3 will present the results obtained from the analysis and the related discussion; the last section is the conclusion and implications for future development.

## 2 Research method

### 2.1 Theoretical concept and the structure of analysis

Sustainable development is referred to long-term continuous development which satisfies current and future human needs. Especially the current needs of human beings should not hamper the demand of future generations [[36], [37], [38]]. Studies have described economic, environmental, and social sustainability are the three most essential pillars for analysing the sustainability of any industry [39,40]. Modern agriculture industries have multiple purposes contributing to and supporting the three aspects of the economy, society, and environment [12,41,42]. However, it isn't easy to conduct a comprehensive total quantitative analysis of all these three factors together to estimate sustainability. The evaluation of these dimensions is difficult to be structured by using one method which can normalise all their standards.

Tongliao APE has special conditions, such as a high-density population and fragile ecological environment, and crop planting and livestock raising are conducted simultaneously. Therefore, the cost-benefit analysis is much more suitable for analysing the economic, environmental, and social factors. It solved the difficulties of qualitatively analysing the feasibility of the project in different aspects to estimate the economic sustainability by calculation of net benefit (net profit), benefit-cost ratio (BCR), and internal rate of return (IRR). To estimate the environmental effect, the cost-benefit analysis is always employed by combining the compensation method in the beef cattle farming industry [43], which can quantify the sustainability of agricultural production. It is more explicit about structuring economic, social, and environmental input (cost) and output (benefit) into a complete equation. However, in previous literature, most studies focused on only one aspect of sustainability [44,45] and did not apply the cost-benefit method comprehensively, which focuses on the APE's sustainability.

**Fig. 2:**
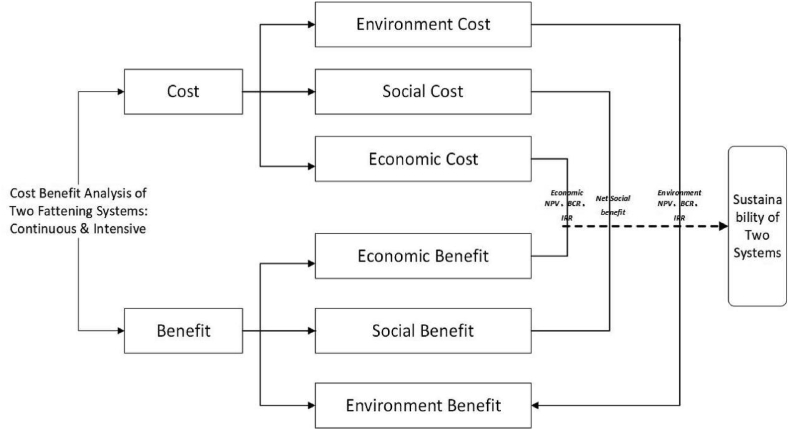
Structure of cost-benefit analysis.

In this paper, we analyse the impact of climate change mitigation (carbon emission) and reduction of pollution resource utilisation method by using net profit value. Social cost-benefit analysis has been applied to consider both internal and external impacts on social welfare, to analyse the social sustainability of the beef cattle farming industry [46]. We measured the cost-benefit analysis based on an economic evaluation to examine sustainability. We constructed the economic benefit by adding feed cost-saving with cattle fattening and breeding income. The economic cost is the combined cost of regular cattle farming and crop planting for the feed supply. The environmental cost of the beef cattle fattening system is mainly from waste treatment. The environmental benefit is primarily reducing air pollution from straw burning by feeding the cattle on straw. The social cost of the cattle fattening system is zero because the social structure has a negligible impact on beef cattle fattening in Tongliao APE. Beef cattle farming brings social benefits to local farming households as public investment improves and reduces the social risk of unemployment. Since crops such as corn and wheat straw are all used as feed for beef cattle in the Tongliao APE area, a large amount of cost-saving from the concurrent business of crop planting and beef cattle farming. The detailed analysis structure and the component of cost and beef cattle farming are described in Fig. 2.

### 2.2 Data and Source

**Fig. 3:**
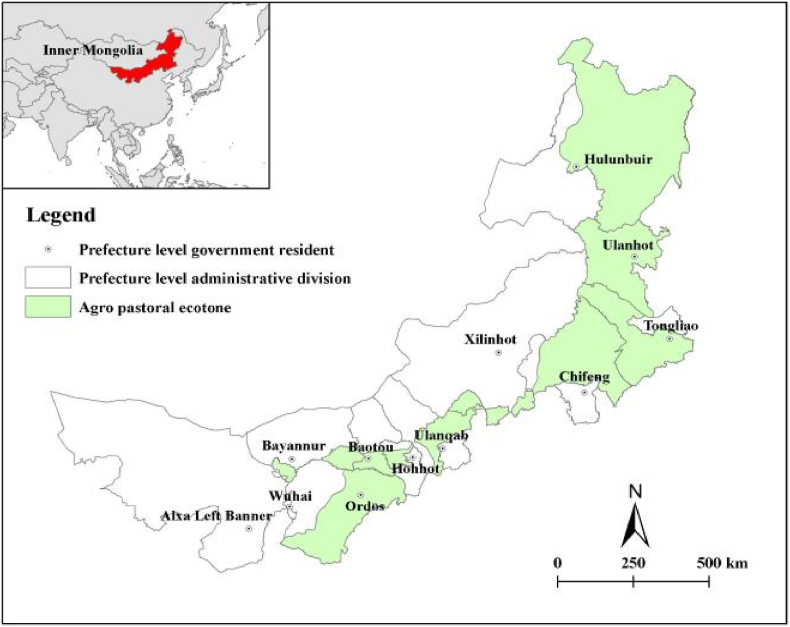
Location of Inner Mongolia agro-pasture ecotone (APE).

This study focuses on the Tongliao Inner Mongolia APE of China, a representative APE that is the joint area of the Northeast and Inner Mongolia Plateau (Fig. 3). To reach the research objective, we considered the two most representative cases to conduct the analysis. Both exemplify the current conditions of the beef cattle industry in Tongliao APE. The six years of data were obtained from 52 households in each village through the survey in 2013–2015 and 2019–2021.

**Fig. 4:**
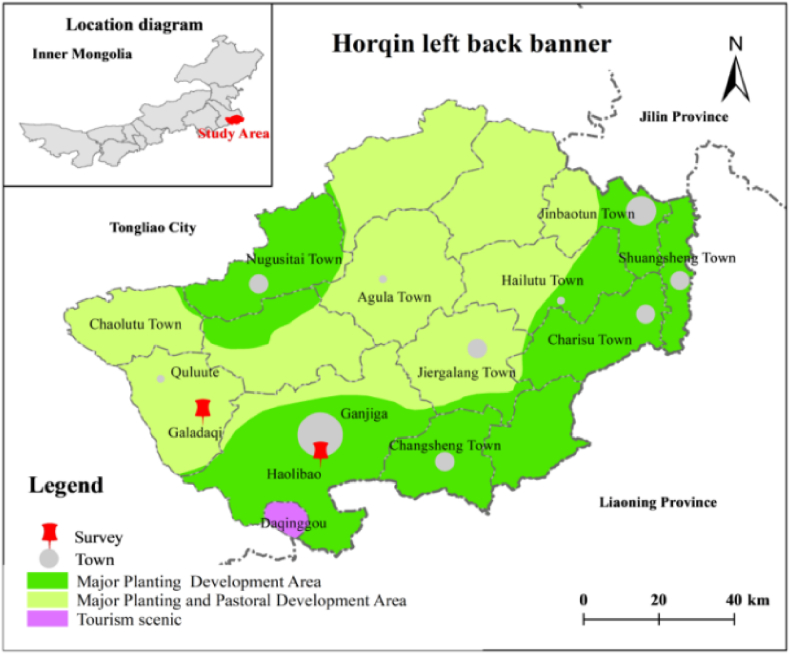
Location of survey area of Tongliao.

The data were obtained from two villages, Galadaqi and Haolibao (Fig. 4). Both villages conduct the concurrent business of beef cattle farming and crop planting. Each household was countered as one production unit. The questionnaire survey was conducted from 52 households in each village, in a total of 104 producers' data was collected in two villages. Each village has data sets in six years with 312 samples and two villages for 624. During survey tracking method was adopted to avoid unreasonable deviation, which means the same households were investigated each time. Galadaqi is the most representative village, which adopts continuous fattening. Haolibao village adopts the intensive fattening system and has the most considerable beef cattle quantity in the Tongliao APE area.

The summary statistics of household data of these two villages are provided in Table 1. The general data of Tongliao city for cattle farming households were also presented in the table. Comparing the data of our survey and the Tongliao Statistics Yearbook, a high representative of the survey data is related to the total beef cattle industry in Tongliao APE. The rural education level in Tongliao is an average of 7.1 years, which is close to our survey data. According to the survey of the Tongliao Beef Cattle industry, household cattle farming number, beef cattle farming numbers are between 10 and 100 for an average household.

**Table 1:** Descriptive statistics of household data of Haolibao and Galadaqi villages.

Name	Measures	Mean	Median	Min	Max	SD	N
Galadaqi Village	Labour number (people)	2.22	2	0	5	0.76	312
	House owner's education (year)	6.12	6	2	11	2.08	312
	Household beef cattle number (head)	28.88	24	0	155	22.48	312
	Farming Land per household in Mu (Mu = 0.067 ha)	56.63	60	0	105	21.79	312
	Planting harvest (ton)	21.94	20	0	55	11.26	312
	Planting Cost (Yuan)	15,681	13,040	2573	59,400	10,362	312
	Expenditure on beef cattle farming (Yuan)	46,626	12,519	260	766,700	92,136	312
	Income from Cattle Sales (Yuan)	74,157	45,450	0	975,000	103,114	312
	Income From Crop Sales (Yuan)	8565	1687	0	75,000	13,145	312
Haolibao Village	Labor number (people)	2.24	2	2	5	0.63	312
	House owner's education (year)	6.83	7.5	3	8	1.41	312
	Household beef cattle number (head)	60.49	41.5	5	320	51.21	312
	Farming Land per household in Mu (Mu = 0.067 ha)	39.23	35	8	100	18.20	312
	Planting harvest (ton)	17.93	15.875	0	60	11.99	312
	Planting Cost (Yuan)	15,342	13,475	1650	77,400	9097	312
	Expenditure on beef cattle farming (Yuan)	448,871	279,712	240	2,448,768	469,072	312
	Income from Cattle Sales (Yuan)	400,066	272,500	0	3750,000	539,449	310
	Income From Crop Sales (Yuan)	1053	0	0	65,000	7501	312

We considered the cattle, feed purchasing, and labour costs for the cost-benefit analysis. In Galadaqi village, the purchasing cost varies for all three types of calf, cow, and bull due to the cow breeding system. However, in Haolibao village, the only purchasing cost is for feeder cattle because there is no breeding cost due to the intensive fattening system. The feed purchasing cost is for additional feed purchasing; feed from self-planted crops is not included. Both villages purchased concentrated feed and coarse fodder but no feed additive. The labour cost included the hired labour cost for each farming sector. New purchased agriculture machinery (tractors, feed mixers, and bulldozers for feed processing, supply, and moving) are used in cattle farming and crop planting. Therefore, it is counted as the cattle farming cost to avoid repetition.

Similarly, the depreciation on fixed assets is also accounted for once for the cross-sector fixed assets. Survey results indicate that dung pollution, air pollution (bed smell), and water waste are the leading causes of pollution caused by the cattle business, which is considered an environmental cost. The cleaning cost of dung is assumed to be the environmental cost of dung pollution. Air pollution is estimated by the average annual cost of deterring the bad smell consumed by each household. The average percentage of water waste is calculated for each household, which varies in both villages. About 30% of the water is wasted in Galadaqi's village, while 40% is wasted in Haolibao village of the total water used for beef cattle farming.

In both villages, cattle farming benefits mainly from cattle sales. Due to different fattening systems, the Galadaqi household generates income from calf, cow, and bull sales and produces a certain amount of cheese to sell. But Haolibao's household generate their income from selling cow and bull. The organic fertiliser fermented by cow manure utilisation rate in both villages is high, which brings a certain amount of income. The net saving from planting corn and using straw as feed brings income to both villages' households as the feature of APE. However, the Galadaqi has more fields for planting as compared to Haolibao. Subsidies, and payments, related to cattle raising are also considered a benefit. In addition, the discount on agricultural insurance is also included.

### 2.3 Model specification

1) Cost-benefit analysis model

(1) NetProfit=Profit=TotalRevenue(TR)–TotalCosts(TC)=TR–VariableCosts(VC)–FixedCosts(FC)

The net profit represents the present value of the difference between the total cost and revenue. Where TR is the current value of the benefit, TC represents the total cost, which includes only variable cost (VC), and total cost does not include the fixed cost (FC). A higher net profit indicates more efficient and economic adaptation of the projects. In other words, the net profit improvement shows that the project's economic sustainability is improving. It can be used as an indicator of a dynamic analysis project's sustainability in the past.

(2) BCR=TR/TC

(3) Netprofit+environ=Netprofit–EnvironmentalCosts

(4) Netprofit+environ+social=Netprofit+environ–SocialCosts

The BCR is the ratio of the present value of benefits and the present value of costs. Where TR is the total revenue, and TC is the total cost. BCR is the benefit-cost ratio. The benefits and costs are each discounted at a chosen discount rate. If the ratio is greater than 1, the project is profitable. The BCR of past years can indicate the economic sustainability of the agricultural project.

## 3 Results and discussion

### 3.1 Cost-benefit analysis

1) Cost comparison of Cattle Farming

**Fig. 5:**
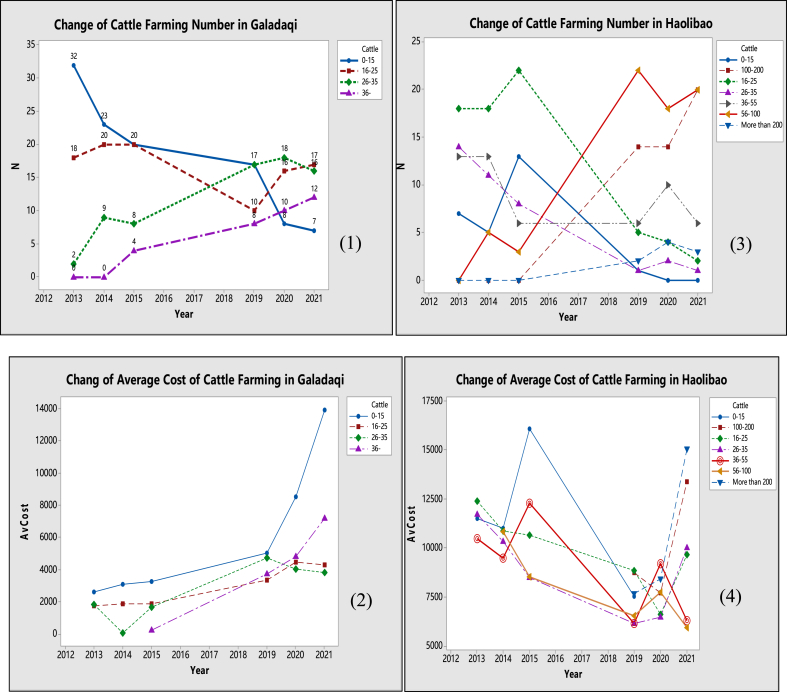
Change in farming cost of beef cattle in two villages 2013–2021 unit: Heads & Yuan.

Fig. 5 presents the change in cattle farming numbers and the average cost of cattle farming in both villages (Galadaqi and Haolibao). Quadrant diagrams 1–4 were drawn based on survey data from Galadaqi and Haolibao. These two villages adopt different approaches: continuous fattening (in Galadaqi) and intensive fattening (in Haolibao). The results show various features.

(Made By Author Based on the Survey Data)

Quadrant diagram (1) of Fig. 5 indicates that the number of cattle farming in Galadaqi village is increasing from the year 2013 to the year 2021. But the continuous fattening approach requires more attention to feeding, breeding, and fattening. In Galadaqi village, the maximum farming number of cattle is 44 heads. The cost of farming in Galadaqi is shown in quadrant (2). The cost of farming is on an increasing trend. However, the rising cost rate surges tremendously in the fewer animal farms compared to more animals. The principle of economy of scale has played a particular role in the cost change of beef cattle farming in the continuous fattening approach. It is inconsistent with the previous literature pointed out that the continuous fattening system has disadvantages in total cost saving [12,[20], [21], [22], [23], [24], [25]] as the cost rate surges faster with fewer cattle numbers. However, in quadrant (2), the result also indicates that the cattle number ranges from 16 to 25 and 26–35 maintain a more stable cost rate than having cattle of 0–15 and more than 36. It suggests the importance of scale economy for the beef cattle business. But in addition, it also indicates that there is a point of diminishing return for the continuous fattening approach of beef cattle farming in Tongliao APE. It argues that the cost change for beef cattle fattening in Tongliao APE is not a static pattern as the cost continuously decreases by increasing cattle. The resource of the labour force, farming land, and feed crop planning also determine the cost of beef cattle farming. According to the survey data, the number of 16–25 cattle is the most efficient range in the cost-saving in Galadaqi village.

The data of Haolibao village showed much stronger fluctuation and instability in the number of cattle farming and farming costs compared with Galadaqi. In quadrant (3), from 2013 to 2021, the household's number of cattle 0–15, 16–25, and 26–35 dropped substantially. This also follows the same previous research results on most beef cattle farming, which tends to approach the intensive fattening system [18, 19]. The households farm had 56-100, 100–200 heads of beef cattle, and their number increased tremendously. The households with more than 200 heads of beef cattle also increased from 0 to a maximum of 4. In quadrant (4), the cost of beef cattle farming in Haolibao is decreasing. The farms with less than 100 heads of cattle show a much faster decrease in farming cost than the farms with more than 100 heads of beef cattle, which indicates that the number of heads also restricts the economy of scale for the intensive fattening approach. According to the survey, households with more than 100 heads of cattle are facing much higher pressure on feed supply and workers' employment. Due to the imperfect information on the feed market and the difficulties of purchasing feeds from other provinces, the average feed cost per head of cattle is much higher for households farming more than 100 heads. In some cases, it is three times higher. In addition, from 2020 to 2021, one factor of increasing cost is a new cycle of beef farming as; households had many new feeder cattle purchased in 2020. Another critical factor is the Covid-19 pandemic, which also caused an increase in the price of feeds (soybean and corn).

In both villages, beef cattle farming households increased in the past years. The increasing rate of cattle farming is much higher in Haolibao than in Galadaqi. It could be due to intensive fattening, which usually requires a large number of beef cattle to reach the level of the economic scale [14, 26]. In Galadaqi, the increasing trend is more benefited from the development of the local economy and the cluster effect of the local beef cattle industry. In addition, about 10% of the feeder cattle of Haolibao are supplied from Galadaqi. Better and accessible selling opportunities in the local market motivated beef cattle farming as the cow breeding system (continuous fattening system) in Galadaqi. However, the number of beef cattle farming is limited in Galadaqi village due to the lack of young farmers and labourers.

Based on the unique feature of Tongliao APE (specific crop supply for feeds, more labour supply compared with pasture area, and animal husbandry tradition), both villages' cost of beef cattle farming may face the problem of efficiency over certain levels, and the price will increase seriously. Compared with Haolibao, cattle farming costs are rising overall in Galadaqi (Fig. 5- Quadrant diagrams 2 and 4). However, it is not enough to conclude that the intensive fattening system is more efficient and sustainable than the continuous fattening system in cost management, therefore comparison of both system is essential. In addition, our new findings showed that the different numbers of beef cattle might produce different patterns of cost change.

**Fig. 6:**
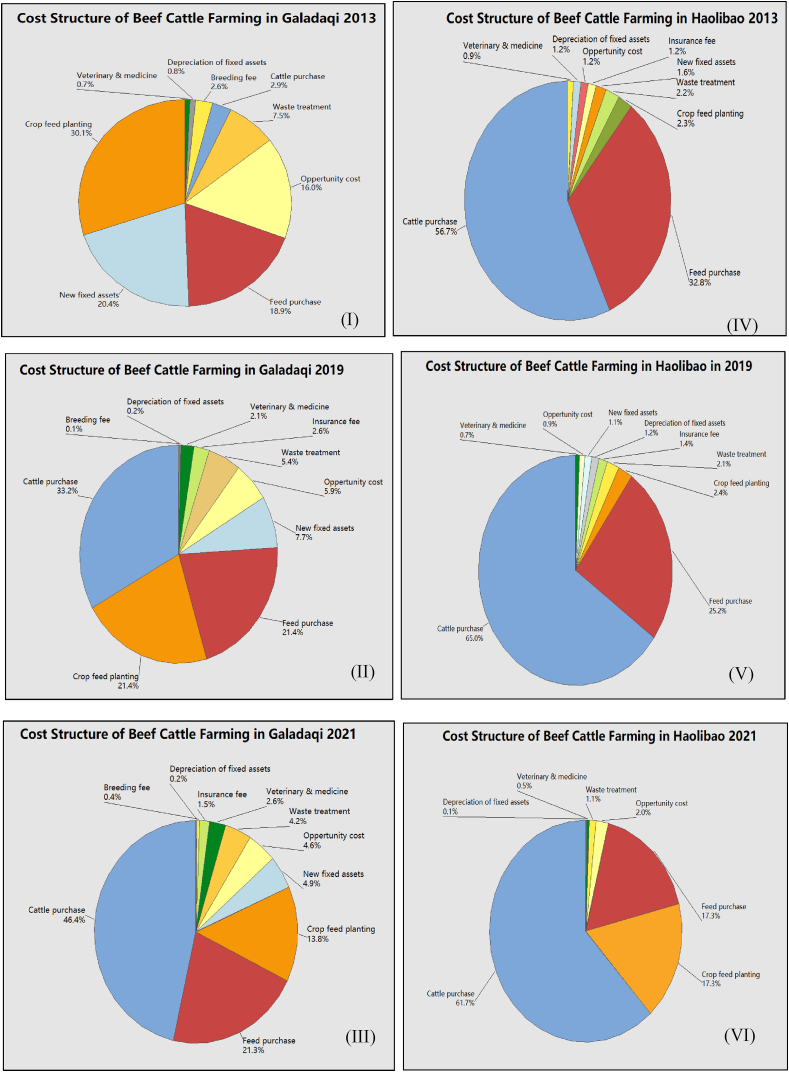
Change of cost structure of two villages 2013–2021 unit: %.

According to the survey data, we draw Fig. 6 (I -VI) to illustrate the average cost structure of the two systems adopted by Galadaqi and Haolibao from 2013 to 2021. Results indicate that the cost structure of Haolibao (Quadrant diagram IV, V, VI) is less changed from 2013 to 2021. In Haolibao, the first, second, and third most significant share of the total cost is cattle purchase, feed purchasing, and crop plantation for the feed of total cost, respectively. In Haolibao, the cost structure is highly associated with the nature of the intensive fattening system, as the farming cycle is a short-term fattening cycle and highly dependent on the number of animals and feed supply. In comparison, Galadaqi village has more changes in the cost structure (I-III). In 2013, the order of cost-share was the crop feed planting, new fixed assets, feed purchasing cost, and opportunity cost, respectively. Because of the continuous fattening, the cost of cattle purchases can be saved. In 2013, the cattle purchase cost only accounted for 2.9%. In addition, a continuous fattening system saves much more feed purchasing costs than the intensive fattening system. In the last two years, the cattle purchasing cost increased, accounting for the largest share of the total cost. Therefore, the smaller calved cow number in Galadaqi makes the demand for feed much less than in Haolibao's fattening systems. In both scenarios, crop planting still contributes substantially to the development of the beef cattle industry, and agro-pasture ecotone (APE) areas provide a massive supply of feeds. However, this contribution is more significant in Galadaqi village because farmers can balance cattle farming and crop planting in better conditions. The above analysis was not conducted in the previous research on compassion between two systems.

(Draw by Author according to Survey Data)

Meanwhile, in both villages, the opportunity cost of beef cattle or sheep proves that the income from beef cattle farming provides higher benefits. Cattle farming has a higher marginal return than traditional farming activities like sheep farming. Fig. 6 indicates that the nature of APE strongly influences both fattening systems adopted by these two villages regarding the cost of beef cattle farming. Especially that crop supplement to the feeds provides a substantial cost saving, promoting the area's economic sustainability. In comparing both systems, the price changes easily influence the total cost of the intensive fattening system in the feeder cattle market. The continuous fattening system which Galadaqi adopts is much more stable as the share of high costs is more evenly separated. This finding is consistent with the previous literature that a continuous fattening system may be more efficient in cost-saving for the Inner Mongolia APE [[33], [34], [35]].

2 )Benefits comparison of Cattle Farming Systems

**Fig. 7:**
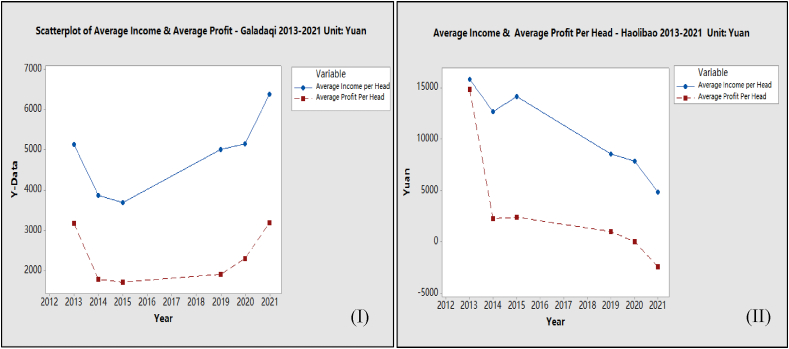
Change of the benefit as the income of beef cattle farming in two villages 2013–2021 unit: Yuan.

The beef cattle farming industry includes economic, environmental, and social benefits. The economic benefits are mainly from the income of selling cattle, which provides for selling milk and cheese and renting machines. In addition, crop planting also benefits in terms of saving purchase costs. The environmental benefits mainly consider using crop straw as beef cattle feed, which can reduce carbon emissions. The carbon emission estimation is based on the carbon sink transfer rate of 18.13. Fig. 7 (I, II) shows the average benefit of beef cattle farming in each household. Results indicated that the income and net profit of beef cattle farming in Galadaqi increased from 2013 to 2021 (quadrant I). However, the income and net profit of Haolibao is decreasing (quadrant II). Meanwhile, the average profit shows significant differences from 2013 to 2021. The continuous fattening system was more efficient. Households with better utilisation of local crops and with a specific range of animals may have this outcome. Previous findings on high return of continuous fattening when less adopting high technology and better feed supply [33] align with this result.

(Draw by Author according to Survey Data)

Results also suggested that the intensive fattening system, which requires a large amount of initial input in fixed assets and feeds purchase, may affect the net profit. The development of an intensive fattening system on technology and feed supply has not yet reached the optimum output level. In Haolibao, local harvesting is difficult for feeding purposes to meet the vast requirement of a large head number of cattle. In addition, the intensive fattening system with a high number of cattle may also meet the time lag of cattle selling which may affect the income and profit, which is another drawback.

3 )Cost-Benefits Analysis Results

The result of the cost-benefit analysis is shown in Table 2. The obtained results indicated the net profit of Galadaqi is much more stable as the continuous fattening system fully utilized the local resources and contributed to the stable TR and RC. The BCR of Galadaqi is also more than 1.5, indicating a substantial cost and benefit structure.

**Table 2:** Net profit and BCR of Two Villages.

Village Name	Year	Average Heads	TR	TC	NET PROFIT	BCR
Galadaqi	2013	13	4771.28	1817.73	2953.55	2.62
	2014	17	3341.82	1798.79	1543.03	1.86
	2015	18	2965.95	1589.05	1376.90	1.87
	2019	35	3024.91	1869.23	1155.68	1.62
	2020	43	2888.70	1694.67	1194.03	1.70
	2021	47	3323.83	1712.37	1611.46	1.94
Haolibao	2013	27	14746.23	926.40	13819.83	15.92
	2014	33	10948.71	9009.85	1938.85	1.22
	2015	26	11362.34	9449.82	1912.52	1.20
	2019	84	5176.22	4582.04	594.17	1.13
	2020	93	4396.58	4400.49	−3.91	1.00
	2021	100	2541.42	3791.69	−1250.27	0.67

TR: Present value of Benefit; TC: Present value of Cost; NET PROFIT: Net present value (RMB).

PV: present value was calculated as the discount of 7.5% which is the basic rate of bank saving.

The net profit and BCR of Haolibao are more substantial but unstable than Galadaqi. In 2021, the sharp drop in benefits was due to the heavy rain, which destroyed the local crop in the Haolibao region. In addition, the feed price escalated by more than 75% in the local market of Tongliao due to the Covid-19 pandemic. Consequently, local households feeding cost burden increased drastically. The cost-benefit analysis results indicate that in 2013–2021, the continuous fattening approach was more sustainable in terms of cost-saving and profit return than the intensive fattening approach. The number of cattle also plays an essential role in controlling cost and risk management due to the feed supply. In contrast, the intensive fattening system was more profitable in those years when the cost of production was more stable.

## 4 Conclusion

Every household is involved in this area's beef cattle farming industry, which brings many advantages to the local farmers. Local farmers adopt different management methods according to the local natural conditions. This study tries to determine the best fattening approach using the cost-benefit analysis of two different fattening approaches. Results indicate that the beef cattle farming industry is rapidly developing in APE areas, and the intensive fattening approach is much more complex, which needs proper attention related to veterinary health care, high investment, and advanced technology during the fattening cycle but requires less land and the farming cycle is short [36]. Cattle purchasing costs contribute the most significant share of the total cost in this system. But intensive fattening systems were more profitable in those years when the production cost was more stable. Previous research has proved that the economic benefits of the continuous fattening system are much more stable and efficient due to the local condition of the agro-pasture ecotone area and the properly utilized local resources, and the BCR ratio (1.5) indicates a substantial cost and benefit structure. It could be because the farmers only consume 2.9% of the total expenditure on animal purchasing in a continuous fattening system. From 2013 to 2021, the net profit of beef cattle farming under this approach increased in general, but the net profit under the intensive fattening system decreased substantially. These are the new findings of our research.

In both fattening systems, the number of animals also plays a vital role in cost control and risk management according to the feed supply of the local area. It is necessary to consider the optimal numbers of cattle raising when adopting these two systems. In addition, a continuous fattening system saves much more on feed purchasing costs than an intensive fattening system. Therefore, around 70% of the local farmers adopt the continuous fattening technique to raise their animals [5, 47]. In contrast, only 30% of farmers of Tongliao APE adopt the intensive fattening approach. Most households with less than 100 cattle animals in intensive fattening systems bear a faster decrease in production costs, but their average income and profit also decreased quickly. The continuous fattening approach is in opposition as the average cost of cattle numbers between 16 and 35 increased slowly, but the average income and profit increased fast. Overall, the continuous fattening approach was more sustainable in terms of cost-saving and profit return than the intensive fattening approach, as it reflected the disparity in net profit and BCR between these two systems. This finding has broken the preconception that intensive agricultural production is always more efficient than traditional household farming methods [26, 27]. Result provides evidence that income from beef cattle farming truly benefits farmers, and participation in the beef cattle farming industry has a higher marginal return than traditional farming activities, but selecting the optimal production method matters for sustainable development outcomes. The analysis of the sustainable production of the fattening system can provide a valuable reference to the policymaking on economic development and environmental protection in the local area. Eventually, the policy should provide more support to the households in the continuous fattening approach in land utilisation for maintaining the self-sufficient output of feed under the environmental and resource restriction of Tongliao APE, meanwhile in the breed improvement for maximising the unit output of beef cattle farming. It is also necessary to optimise the policy support on the intensive fattening system to shift the traditional single financial aid such as bank loans into more technological supports and training to reach the real economic of scale on production and environment mitigation. The beef cattle industry will benefit the local people's lives and promote environmental protection in the long term. This study can also be a good reference for other developing countries where farmers are getting no proper training on production and management practices of beef cattle farming. In addition, understanding both production systems will lead to an enhanced beef cattle industry, especially if the area's local geographical and climatic needs are considered.

## 5 Limitations

This study considered the social cost of the cattle fattening system to be zero because the social structure has a negligible impact on the beef cattle fattening system in the local area. It is not easy to accurately evaluate the social benefits in the monetary form. In the current research, most of the social benefits are intangible. But certainly, beef cattle farming brings social benefits to the local household, as the increasing trend in public investment in the beef cattle industry reduced the social risk of unemployment. Because of the large rural population, the cattle industry can absorb the surplus labour force. Usually, water wasted on a feedlot, cleaning, and water-feeding waste is annual water waste. But it was impossible to calculate the actual water wastage ratio due to the unavailability of research in China, On the water waste of beef cattle and each household's different sizes of feedlots and water-using habits. Therefore, this study considered the average percentage of water waste in total water use.

Consequently, it is complex to transform the real social benefits into a monetary base. It always showed arbitrary results on estimation. Therefore, to avoid the risk of overestimating social benefits, this study accepted the high potential of positive social benefits but did not count into the total benefit. In addition, this study ignored the calf sale because most households adopted the intensive fattening system in Haolibao village, and the calf-born rate is shallow. But it has some impact on the household's income. This study is based on a limited period of 8 years, while the beef cattle industry is a long-term development process. Detailed studies on different contaminants in cattle feed are needed to understand the benefit distribution.

## Funding

This research work was supported by the National Key Research and Development "intergovernmental International Co-operation in Science and Technology: Key Project Sino- Mongolian Agriculture and Animal Husbandry Supply Chain Collaborative Research (Grant No.: 2021YFE0190200).

## Footnotes

# According to the length of fattening time, it can be categorized into two types of fattening: long term (more than 9 months) and short term (3–4 months). In Tongliao, long term fattening is normally taken [6].
